# Metabolomic strategies for the identification of new enzyme functions and metabolic pathways

**DOI:** 10.15252/embr.201338283

**Published:** 2014-05-14

**Authors:** Gareth A Prosser, Gerald Larrouy-Maumus, Luiz Pedro S de Carvalho

**Affiliations:** Mycobacterial Research Division, MRC National Institute for Medical ResearchLondon, UK

**Keywords:** enzyme annotation, mass spectrometry, metabolomics, pathway discovery

## Abstract

Recent technological advances in accurate mass spectrometry and data analysis have revolutionized metabolomics experimentation. Activity-based and global metabolomic profiling methods allow simultaneous and rapid screening of hundreds of metabolites from a variety of chemical classes, making them useful tools for the discovery of novel enzymatic activities and metabolic pathways. By using the metabolome of the relevant organism or close species, these methods capitalize on biological relevance, avoiding the assignment of artificial and non-physiological functions. This review discusses state-of-the-art metabolomic approaches and highlights recent examples of their use for enzyme annotation, discovery of new metabolic pathways, and gene assignment of orphan metabolic activities across diverse biological sources.

## Introduction

The functional annotation of the wealth of genetic information generated during the genomic era is currently considered one of the grand challenges in molecular biology. Although bioinformatics and modelling have contributed considerably to the functional assignment of proteins, significant portions of sequenced genomes (between 30 and 40% of genes by a recent estimate 1) remain unannotated or are ascribed a putative function. Accurate genome annotation is essential for developing a comprehensive and detailed understanding of cellular physiology and is therefore a primary concern in almost every avenue of biological research.

*In silico* sequence homology-based methods have been the driving force behind most genome annotation endeavours to date 2. Despite their strengths in automation and sample throughput, such techniques are unable to identify the functions of novel gene sequences that have little to no homology with pre-existing database entries or may lead to the misannotation of gene products that share very high homology but catalyse fundamentally different reactions. Gene misannotations in particular are a prevalent consequence of automated *in silico* methods, and the propagation of such misannotations is a serious and growing threat to the accuracy and reliability of genome and protein databases 3–5. Automated genome annotation remains the only viable option to efficiently process genetic sequences at their current rate of influx, although a more comprehensive and experimentally determined understanding of the relationship between primary sequence and function is clearly required in order to improve annotation accuracy.

**Figure 1:**
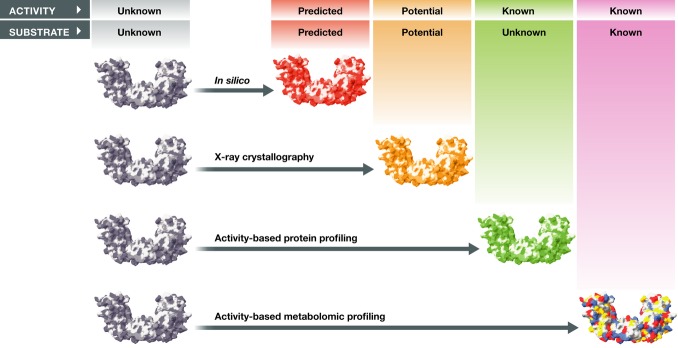
Approaches used to uncover the function of orphan enzymes

A significant proportion of unannotated or misannotated genes encode enzymes, the catalytic activity of which is usually fundamental to their physiological function. Techniques that can directly exploit or monitor the native activity of a candidate enzyme are therefore powerful tools in accurate functional assignment of unannotated gene products: several approaches that have historically been used to annotate enzyme function, as well as newly developed techniques, are described in Fig 1 and Table 1. However, many activity-based assays—for example, those performed *in vitro* with purified enzyme preparations—require at least a prior basic knowledge of the type of reaction catalysed by or substrate specificity of the candidate enzyme and therefore lack broad applicability. Furthermore, testing individual putative substrates on a case-by-case basis is time-consuming and expensive and relies on the native substrate being commercially available. Alternatively, screens performed *in situ* in the host organism are less biased as they evaluate enzyme activity within a physiologically relevant milieu. Forward and reverse genetic screens, for example, are fundamental tools in uncovering the function of unknown gene products, but ultimately rely on the emergence of observable phenotypic traits for successful gene assignment, which does not occur for many mutants 6,7. An activity-based proteomics approach (activity-based proteomic profiling; ABPP) has been recently used to identify class-specific enzymes within a complex mixture (including *in situ* in cells) in an unbiased manner using covalent active-site-directed probes 8. Its potential as an enzyme function discovery tool has been illustrated by the successful assignment of mechanistic class and function to previously unannotated enzymes that lack sequence homology with canonical members of their enzyme class 9,10. Despite its dependence on unique synthetic chemistry tools and limited scope in native substrate identification, this technique demonstrates the usefulness of physiologically relevant sequence- and phenotype-independent tools in modern functional genomics.

**Table 1:** Strategies for enzyme function discovery

Techniques available	Enzyme/genetic requirements	Purposes	Advantages	Inconveniences	Key technologies
*In vitro* activity-based metabolomic profiling	Purified, homogeneous enzyme	Track enzyme-induced changes in a complex metabolite extract	High throughput (hundreds to thousands of metabolites can be screened).	Enzymes have to be purified to homogeneity.	Protein purification
			*Physiologically relevant library of substrates and co-factors.*	Host organism or related species has to be cultured.	LC/GC/CE-MS
			No *a priori* knowledge of the types and number of substrates and products involved.	Recombinant expression might lead to loss of native partner or post-translational modifications required for activity.	NMR
			No *a priori* knowledge of the type of chemistry catalysed.	Substrates might not be present at quantifiable levels in molecular extract.	Libraries of spectral data
			Direct identification of potential substrates and products.		
*Ex vivo* metabolomic profiling – genetically modified/chemically treated organism	None or verified genetic knockout/over-expression strain of organism of interest	Identify one enzymatic reaction or pathway that is disturbed upon deletion/alteration of levels of a particular enzyme	High throughput (hundreds to thousands of metabolites can be screened).	Host organism or related species has to be cultured and genetically tractable.	Genetic manipulation LC/GC/CE-MS
			No knowledge of the types and number of substrates and products involved required.	Candidate substrates and products might constitute secondary effect changes.	NMR
			No knowledge of the type of chemistry catalysed required.	Levels of substrates/products might be tightly controlled and not change.	Libraries of spectral data
			No enzyme purification required	Chemical with a clear phenotype must be available.	
			Preservation of native enzyme partners and post-translational modifications.		
Activity-based protein profiling	None	Track activity of a specific class of enzymes towards a probe	High throughput (several dozen enzymes can be screened).	Highly selective and specific probe needs to be synthesized.	Chemical probe
			Identifies active enzymes.	Identification of physiological substrates needs to be carried out subsequently.	Gel electrophoresis
			Highly specific for the chemistry and enzyme class to which the probe has been developed.	Host organism or related species has to be cultured.	Imaging
			No enzyme purification or genetic modification required	Active enzyme of interest needs to be identified.	Protein identification
			Preservation of native enzyme partners and post-translational modifications.		
Computational enzymology	High-resolution structure	Identification of putative substrates, products and intermediates based on structural determinants	High throughput *in silico* approach can be applied to any enzyme type.	Relies on strength of ligand docking software and accuracy of crystal structure.	Docking
			No *a priori* knowledge of substrate specificity or type of chemistry catalysed required.	Identified compounds might not exist in the host organism.	Virtual libraries
					Computation
X-ray crystallography	Purified, homogeneous enzyme	Identify co-purified small molecules associated with purified enzyme	Tightly bound ligands can directly lead to the identity of substrates/products/intermediates.	Enzymes have to co-purify with a tightly bound metabolite.	Protein purification
	High-resolution structure			Enzymes have to be crystallized and the structure has to be solved at sufficiently high resolution.	Crystallization
				Bound ligand structure has to be determined.	Structure determination
				Bound ligand might not be present in the host organism or be related to the native function.	

NMR, nuclear magnetic resonance; GC/LC/CE, gas or liquid chromatography or capillary electrophoresis.

Metabolites constitute the substrates and products of enzymatic reactions, and the study of the total metabolite pool of a given organism or cell type is known as metabolomics. As metabolites represent the final outcome of gene expression and activity, the metabolome can be perceived as the ultimate readout of the biochemical and physiological state of a cell, that is, a direct link between mechanistic biochemistry and cellular phenotype 11. The concept of metabolomic profiling for assessing cellular or bodily function is not new 12; however, the analytical and computational technologies have only recently become sufficiently powerful and widely accessible to allow routine and unbiased investigations of cellular metabolite pools. With the latest improvements in mass spectrometry (MS), for example, it is now possible to sample hundreds to thousands of unique ion peaks, assign putative (or verified) molecular formula to these peaks, and even extrapolate native intracellular concentrations, all from a small (even single cell analysis 13) quantity of starting material 14,15 (Sidebar A). Due to these recent innovations, metabolomics is rapidly becoming a routine discipline in diverse areas of biological research, including disease biomarker and drug target discovery, drug pharmacodynamics, and metabolic engineering 16–21. The ability to impartially monitor metabolic transformations from a variety of biological sources has also allowed metabolomics to thrive within the context of enzyme function discovery, typically in combination with recombinant genetic and protein expression tools. The focus of this review is the application of activity-based metabolomic strategies in three principal areas of functional genomics: the discovery of novel metabolic functions and pathways, the functional assignment of unannotated genes, and the assignment of gene sequences to orphan metabolic activities.

**Sidebar A: Mass spectrometry for metabolomics**

The diversity and quantity of metabolites that comprise a metabolome varies depending on the organism, and can range from several hundred (bacteria) to several thousand (mammals, plants) unique low-molecular-weight (< 1000 Da) chemical entities 82,83. The relative scarcity of metabolomic investigations, relative to other ‘omics strategies’, is a consequence of the difficulties associated with the unbiased analysis of the wide physicochemical heterogeneity apparent across these compounds. Although procedures for analysing specific chemical classes have been employed for some time (‘targeted’ metabolomics 84,85), the ability to assess a range of classes simultaneously and impartially (‘untargeted’) has only become possible with recent technological progress in certain analytical platforms; in particular nuclear magnetic resonance (NMR) and mass spectrometry (MS). NMR offers advantages through milder sample processing and a more robust quantitative output, but MS-based methods have dominated the field due to their superior mass resolution, sensitivity (atto- to zemptomolar 86), and mass accuracy (sub millidalton 87). Furthermore, MS can be combined with sample separation techniques such as gas or liquid chromatography or capillary electrophoresis (GC/LC/CE) to enhance the detection of individual species. Another major benefit of MS-based platforms is the increasing number and quality of publicly available databases dedicated to mass spectral profiles of authentic standards, enhancing confidence in matching experimental data peaks to molecular structures 88–91. The intricacies of MS function, sample preparation and data analysis are beyond the scope of this review, and have been excellently covered elsewhere 11,88,92.

## Discovery of New Metabolic Functions and Pathways

A significant impediment to large-scale assignation of enzymatic function is our incomplete understanding of primary and secondary metabolism, even among well-studied organisms such as *Escherichia coli*. Consequently, the discovery of novel metabolic pathways is an ongoing effort 22–25. The ability of metabolomics, particularly in combination with stable-isotope probing (Sidebar B), to follow the metabolic fate of target compounds and their flux through specific pathways underlies its utility in the identification of new biochemical reactions occurring within cells. In this context, new pathways and metabolites can be identified without the need for targeted genetic modification or recombinant protein studies, simplifying the workflow and allowing greater flexibility in the conditions and test organisms used. Several approaches to metabolite and pathway discovery using activity-based metabolomics methodologies are outlined below.

**Sidebar B: Stable isotope labelling**

Stable isotope labelling or SIL (mainly with ^1^^3^C and ^1^^5^N) is an essential component of most studies utilizing discovery metabolomic techniques. Two types of information can be uniquely gathered from well-designed SIL experiments: (a) metabolic fate, and (b) metabolic pace.

**Figure 2:**
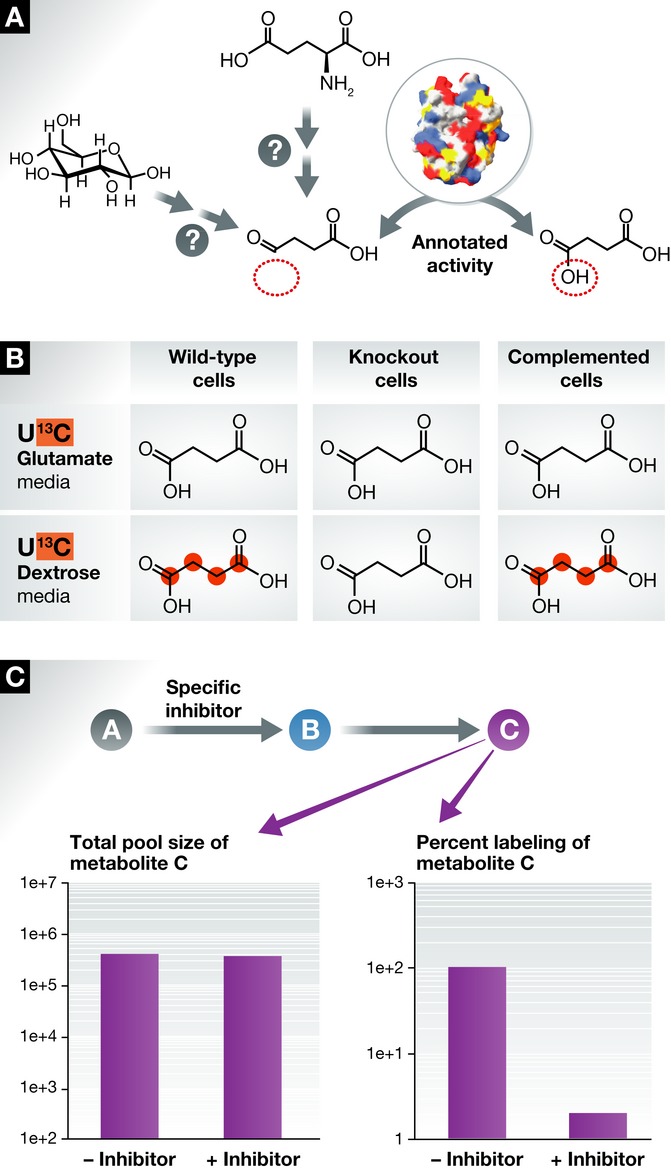
Labelling experiments can probe pathway(s) and the metabolic context of newly identified enzymatic activities (A) An enzymatic reaction is identified by activity-based metabolic profiling or another method. The structure is of *Mycobacterium tuberculosis* CitE 99. (B) Possible outcome of a labelling experiment designed to probe the origin of the carbon backbone of succinate. In this case, as glutamate labelling does not generate labelled succinate, a classic GABA shunt starting from glutamate is ruled out. Labelling with dextrose would therefore indicate a mitochondrial role for this newly identified enzyme. (C) Labelling experiment designed to confirm that the enzymatic activity identified belongs to the pathway described. The specific inhibition of synthesis of compound B leads to no change in the pool size of metabolite C. However, it drastically diminishes its labelling, supporting the relationship between A and C.

Metabolic fate defines the relationship between the reaction identified and its exact position in the metabolic network to which it belongs. This is especially relevant when substrates and/or products of the candidate enzyme overlap multiple metabolic pathways, obscuring the true physiological function of the enzyme. For example, if enzyme X converts succinic semialdehyde (SSA) into succinate, there are at least two known potential metabolic pathways in which this enzyme could serve (Fig 2A). The first is the γ–aminobutyric acid (GABA) shunt 93 which is involved in glutamate and GABA metabolism and is of particular importance in neurotransmitter regulation in the mammalian central nervous system. The second involves detoxification of SSA generated by the α–ketoglutarate dehydrogenase complex under non-optimal Krebs cycle conditions, which can be caused by mutations or environmental stimuli such as oxidative stress 94,95. Therefore, by measuring the extent of succinate labelling following selective supplementation of growth media with either labelled glutamate or a labelled glycolytic intermediate (e.g. dextrose), one can unambiguously define the specific pathway in which enzyme X is involved (Fig 2A and B), and by consequence gain a more detailed indication of the enzyme’s physiological role (e.g. neurotransmitter metabolism versus mitochondrial defect).

Metabolic pace refers to the flux (rate) of biochemical reactions and pathways in living cells (Fig 2C). Time-course experiments with SIL can therefore provide direct evidence for variations in the flux of specific biochemical pathways that have been perturbed by chemical or genetic means. Metabolic rate studies of newly discovered/annotated enzymes are particularly informative when the concentration of a determined metabolite (pool size) does not change considerably upon gene deletion or drug treatment, but the flux through the pathway is affected 96–98.

Our current understanding of central metabolism has been influenced by studies performed in a small number of model organisms under a limited set of test conditions, suggesting that conditionally induced pathways are poorly represented in metabolic charts. Varying growth conditions, or applying abiotic or biotic stimuli to cultures of interest, are therefore useful tactics for the elucidation of cryptic pathways. Farag *et al* recently used a metabolomics approach to delineate several novel isoflavonoid and phenylpropanoid pathways in the leguminous plant species *Medicago truncatula* after stimulation with methyl jasmonate and yeast elicitor, two agents known to influence plant secondary metabolite synthesis 26. The use of stable-isotope tracers in combination with metabolomic profiling allowed the authors to define the precise biosynthetic origin, metabolic pathway, and molecular formula of each identified compound, as well as the complex regulatory patterns governing the expression of each pathway. Many plant secondary metabolites have important pharmacological and biotherapeutic properties, and it is therefore crucial to understand their biosynthetic routes and regulation. Metabolomics-based strategies should have a central role in achieving this goal.

Similarly, new pathways and functions are likely to be found in non-model organisms, particularly those inhabiting unique physical environments—such as thermophiles and halophiles. Importantly, non-canonical metabolic processes that occur in these organisms, or those able to operate under non-mesophilic conditions, are often highly sought after as biotechnological or industrial tools. Stable-isotope probing combined with metabolomics and fluxomics was recently used to uncover an unusual isoleucine biosynthesis pathway in *Geobacter metallireducens* 27 and the formation of a non-canonical (*R*-) stereoisomer of citrate in the Krebs cycle of *Desulfovibrio vulgaris* 28, two environmentally important microorganisms involved in global element cycling pathways. In addition, the long predicted ethylmalonyl-CoA pathway for growth on reduced single carbon compounds was finally demonstrated to exist through the combined application of metabolomics and stable-isotope flux analysis in the methylotrophic bacterium *Methylobacterium extorquens* 29. Understanding how microorganisms are able to convert single carbon compounds into complex biomolecules is of major interest in biotechnology, particularly for biofuel production, and these studies exemplify the potential of metabolomics to uncover hitherto unknown metabolic pathways.

Alternatively, molecular genetics can be used in combination with metabolomics as an effective hypothesis-generating tool to discover new metabolic functions: the disruption of a pathway of interest might lead to the upregulation of alternative pathways to compensate for the lost function(s). For example, despite the essentiality of central carbon metabolism in all living systems, studies have shown that just 4 of the more than 70 enzymes involved in *E. coli* glycolysis and the tricarboxylic acid (TCA) cycle are essential for growth under standard laboratory conditions 30. Such genetic redundancy indicates the presence of alternative compensatory pathways or isoenzymes that operate in the event of primary pathway disruption 31. Nakahigashi *et al* recently analysed the biochemical mechanisms of pathway compensation in *E. coli* strains deficient in primary enzymes of central carbon metabolism 32. One unexpected observation was the lack of a growth defect in a transaldolase-deficient mutant when xylose was supplied as sole carbon source, as transaldolase provides the link between the pentose phosphate pathway and glycolysis. Metabolomic profiling revealed the appearance of a molecular species equivalent to sedoheptulose-1,7-bisphosphate only in the mutant strain, which was further confirmed through genetic screening and metabolic flux analysis and found to be a novel side reaction of 6-phosphofructokinase I (*pfkA*). The combination of genetic tools and metabolomics has also been used to identify a previously unrecognized uridine monophosphate (UMP) degradation pathway in *E. coli* strains that are deficient in a negative feedback regulator of pyrimidine homeostasis 33. In this case, metabolomic profiling led to the identification of not only a novel enzymatic activity (UMP phosphatase), but also a unique ‘directed overflow’ regulatory mechanism involved in pyrimidine catabolism. These examples illustrate the power of metabolomics approaches to delineate both the enzymatic activities and regulatory features responsible for the correct function of metabolic pathways.

## Co-Expression Analysis: Combining Metabolomics with Transcriptomics

The understanding of novel metabolic activities and pathways is incomplete without the identification of the genes responsible. As already mentioned, untargeted investigations of new biochemical pathways can rarely associate metabolic reactions with the enzymes that catalyse them without subsequent genetic intervention or recombinant protein studies. One method that can overcome this is co-expression analysis. This transcriptomics-based method allows the identification of genes involved in a defined metabolic pathway based on their co-expression with genes of known function, through the assumption that genes involved in the same biochemical pathway are co-regulated 34. For example, by comparing transcriptome data sets of a test organism cultured under various conditions, sets of genes that are commonly co-expressed are predicted to operate in a single metabolic pathway. As a stand-alone technique, co-expression analysis has been an instrumental tool in functional genomics, allowing high-throughput genome-wide and inter-species predictions of protein function 35,36. However, protein functional assignment is usually restricted to the overall pathway level, and specific enzymatic functions can rarely be extrapolated from the co-expression data alone. The recent combination of co-expression analysis with metabolomics-based platforms has increased the potential of this approach. This allows the use of changes in metabolomic profiles that correlate with changes in transcriptomic profiles to predict putative associations between genes and defined metabolic functions. For example, this approach has been successfully applied in the assignment of sulfotransferase and glucosyltransferase functions to genes involved in glucosinolate and flavonoid biosynthesis in *Arabidopsis*, respectively 37,38. Importantly, the predicted enzyme functions were validated through subsequent genetic and recombinant enzyme-based assays. Although effective in both pathway discovery and gene assignment, a significant pitfall of this approach is the reliance on a direct and positive correlative relationship between gene expression levels and metabolic activity. As post-transcriptional modifications and regulation of enzyme function have substantial effects on most, if not all, metabolic pathways, many of the causal effects between transcript and metabolite levels may be lost in the resulting data sets.

## Enzyme Annotation

### Activity-based metabolomic profiling approaches

**Figure 3:**
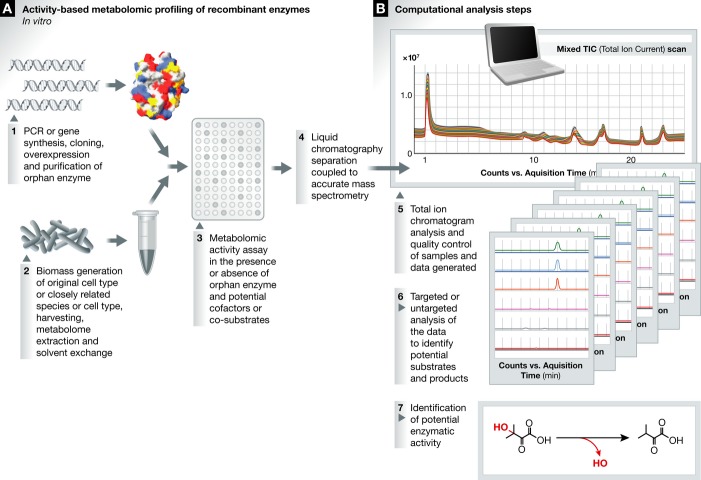
Activity-based metabolomic profiling of recombinant enzymes (A) *In vitro* experimental set-up. (B) Common computational analysis steps.

*In vitro* studies with purified recombinant protein often provide the most definitive proof of a *bona fide* activity for a specific enzyme, as consumption of substrate and generation of product can be monitored directly in an isolated system, free of contaminating species. The disadvantages of this approach are that putative substrates need to be tested individually (or in small batches) and are often not commercially available, or, if so, prohibitively expensive. A possible solution is to engage the metabolome of the parent organism as a substrate library substitute. The metabolome represents a chemically diverse and rich mixture of physiologically relevant compounds, including those that are unknown or not commercially available, and is easily and cheaply obtained in large quantities (depending on the culturability of the parent organism). After the incubation of a recombinant enzyme of interest with a cellular metabolome extract (including any required co-factors or co-substrates), mass changes can be monitored by mass spectrometry to identify putative native substrates, which decrease in abundance, and products, which increase in abundance (Fig 3). Furthermore, reactions are run in near-native conditions (in the absence of metabolite or protein tagging or labelling) and genetic modification of the host organism is not required, simplifying the overall workflow and improving accessibility to non-specialist laboratories and non-model organisms. The identification of enzyme-induced spectral changes among the complex metabolomic profile obtained by MS can be eased by the use of dedicated MS data analysis software, such as XCMS 39. This general approach was first implemented by Saito *et al*, who used capillary electrophoresis (CE)-MS on mixtures of recombinant enzyme and *E. coli* small molecular extract to identify the phosphatase and phosphotransferase activities of two *E. coli* uncharacterized enzymes, YbhA and YbiV 40. Similar workflows have subsequently provided mechanistic insights for unannotated or misannotated enzymes from a variety of functional classes and biological origins 41–44. Several cases of particular interest are outlined below.

The human cytochrome P450 monooxygenase (hP450) family of enzymes comprise more than 50 members and have significant roles in the normal physiological metabolism of a variety of lipids, sterols, vitamins and xenobiotics 45. Their diversity, however, has complicated the functional assignment for each member, and currently more than a quarter of annotated hP450s have unknown functions 46. Guengerich *et al* have extensively characterized the substrate specificity of hP450 enzymes, with successful application of metabolome-based *in vitro* assays in many cases 47–49. One approach they pioneered is the use of stable-isotope-labelled co-substrates in metabolome-based *in vitro* reactions to facilitate the positive identification of substrates and products after analysis by MS 50,51. In this case, the reaction containing cellular extract and enzyme is allowed to proceed in the presence of a 50:50 mixture of ^18^O and ^16^O-labelled oxygen gas, causing the resulting enzymatic product to emit a 1:1 ratio of native (M) and isotopically labelled (M + 2) *m/z*s on a mass spectrum plot. Although this approach is applicable to enzymes that have other common co-substrates, such as ATP, NAD(P)H, or SAM (as long as isotopically labelled versions are available), the prerequisite of knowing the identity of the co-substrate limits its application to putative enzymes of known functional class.

Genetic techniques are powerful tools in functional genomics, allowing target gene disruption and subsequent assessment of the resulting phenotype or metabolic status to infer important information on enzymatic function. However, genetic methods are not always applicable, such as when the candidate gene is essential for growth or the host organism is not genetically tractable. In these cases, *in vitro* metabolomic methods are particularly useful for characterizing enzyme function. For example, although genetic tools are relatively well developed for the human pathogen *Mycobacterium tuberculosis*, the slow growth rate (~20 h doubling time) of this organism makes gene knockout protocols cumbersome. Consequently, we have used *in vitro* metabolomic methods to identify the functions of two uncharacterized *M. tuberculosis* gene products. One is a 2-hydroxy-3-oxoadipate synthase that was previously annotated as an oxoglutarate decarboxylase component of the Krebs cycle oxoglutarate dehydrogenase complex 43 and the other a haloacid dehalogenase superfamily member with glycerol-3-phosphate phosphatase activity involved in the recycling of cell-wall lipid polar heads 44.

### Metabolomic profiling approaches

**Figure 4:**
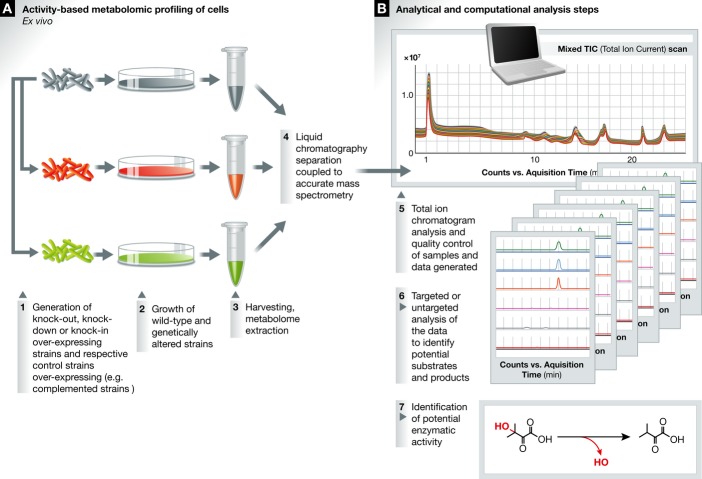
Global profiling of cellular metabolome (A) Activity-based *ex vivo* metabolomic profiling of genetically or chemically modified cells. (B) Common computational analysis steps.

Metabolic enzymes do not function in isolation. Indeed, the core of biochemical network regulation relies upon a multiplicity of complex and dynamic interactions between the various enzymatic players and metabolites involved 52. Both catalytic competency and substrate specificity can be significantly modified via post-translational modifications or allosteric interactions with other biomolecules, according to metabolic requirements 53,54. As such, *in vitro* technologies are generally insufficient, in isolation, to attain an accurate and detailed picture of the physiological function of a candidate gene. Instead, *in* or *ex vivo* approaches are becoming increasingly popular as primary or complementary screens in enzyme function or pathway discovery, with metabolomics at the forefront of these advances. As already mentioned, *ex vivo* metabolomics has long been a major tool in the delineation of cellular metabolic circuitry and is still a key experimental platform in pathway and metabolite discovery 25. However, the lack of integration between metabolomic and genetic data in such workflows largely precludes the ability to unequivocally match gene products with individual chemical transformations. Instead, the application of *ex vivo* metabolomics to conditional knock-down, stable genetic mutants, over-expression strains or to chemical knock-downs of a candidate gene product allows a more precise and specific assessment of the function of a gene in cellular metabolism (Fig 4). This methodology was initially used to analyse gene deletion strains of *Sacchromyces cerevisiae*; whole-cell metabolomes from strains deficient in genes of unknown function were globally compared—by principal components analysis—to those deficient in genes of known function that spanned a variety of metabolic pathways 55. Data sets that clustered similarly provided the necessary evidence to assign a gene product to a specific metabolic pathway, without defining a precise enzymatic function. Improvements in metabolomic data resolution and processing power subsequently allowed Saghatelian *et al* 56 to precisely identify several natural substrates of the mammalian enzyme fatty acid amide hydrolase (FAAH) by comparing the brain metabolomes of wild-type and FAAH null mutant mouse strains, thus providing the first successful integration of *ex vivo* metabolomics and gene function discovery. The succeeding decade has witnessed the application of similar workflows to the discovery of natural substrates and catalytic functions for a variety of metabolic enzymes from a range of cell types/organisms 57–62.

*Ex vivo* metabolomic profiling has seen considerable success in the assessment of enzymes involved in secondary metabolite biosynthesis, in particular non-ribosomal peptide (NRPS) and polyketide synthase (PKS) pathways, the products of which are of substantial biomedical interest due to their pharmacological properties and roles in virulence. In these cases, bioinformatics can reliably predict gene clusters likely to code for NRPS or PKS operons, and subsequent metabolomics of individual gene mutants provides the necessary means of both identifying the final metabolite and assigning each of the chemical steps involved in its biosynthesis to individual gene products 63–65. The strength of using an untargeted metabolomics approach in these situations is evident when considering the myriad of diverse chemical transformations involved in secondary metabolite biosynthesis. Few alternative technologies would have the capacity to simultaneously monitor such a distinct array of enzymatic reactions using a single analytical platform. For example, Schroeder *et al* recently used genetics and metabolomics to identify the catalytic roles of each of the eight gene products responsible for the production of a new siderophore and virulence factor, hexahydroastechrome, in the filamentous fungus *Aspergillus fumigatus* 66. By systematically profiling the metabolomes of individual gene deletion strains, the authors were able to assign P450-like hydroxylation, O-methyl transfer, prenylation, and FAD-dependent carbon–carbon double-bond formation activities to each gene product in the pathway.

In addition to the canonical approach described above, several groups have demonstrated that gene functional assessment by *ex vivo* metabolomics need not require genetic disruption of the gene of interest. This is particularly useful in cases where gene function is essential for cell survival, the host organism does not have readily available genetic tools, or where genetic inactivation of the candidate gene does not cause significant metabolomic changes due to inherent genetic robustness of the test organism 67. For example, Chiang *et al* recently used a mechanism-based inhibitor to inactivate and interrogate the physiological function of an integral membrane hydrolase (KIAA1363) that had been shown to be upregulated in certain aggressive cancers 68. The metabolomic impact of the inhibitor on target cells permitted the identification of 2-acetyl monoalkylglycerol as the physiological substrate for this enzyme. Although effective in this case, the reliance on synthetic chemistry resources and/or known inhibitors of the candidate enzyme will likely limit the broad applicability of this approach. Furthermore, generic inhibitors that target specific functional enzyme classes are likely to have pleiotropic effects on cellular metabolism, complicating subsequent data analysis. Transient over-expression of candidate enzymes in test cell lines *in situ* and subsequent metabolomics has been used by Cravatt *et al* to investigate the function of a series of unannotated human serine hydrolase family proteins 69. Depletion of substrate and overabundance of product in resulting mass spectrum traces allowed the identification of phosphatidylcholine phospholipase activity for one of the test enzymes, ABHD3. As the authors stated, however, care must be taken when interpreting results obtained through this approach, due to possible metabolic artefacts arising from the artificial over-expression system, or the absence of a physiological substrate due to the use of a heterologous host.

## Enzyme Discovery: Assigning Genes to Known Metabolic Activities

The experimental workflows and examples discussed above implemented a ‘reverse genetics’ approach, whereby the coding gene of interest is initially chosen by bioinformatic means and the cognate function subsequently sought with activity-based metabolomics. Although this is a logical process for assigning function to unannotated genes, the reverse process, that is, assigning gene sequences to predetermined orphan metabolic activities or pathways, is largely excluded.

Orphan metabolic activities are an alternative and growing problem within functional genomics, a consequence of many decades of pre-genomics enzymology research, as well as the recent and rapid rise in untargeted metabolomics data accumulation. For example, recent studies found that 30–40% of all enzymatic activities currently assigned an enzyme commission (EC) number are not associated with a defined genetic locus 70,71. Although the methodology is not as well developed as for gene annotation, activity-based metabolomics is beginning to show promise as a valuable platform for de-orphaning metabolic activities, as illustrated by two recent studies. Both of these relied on a ‘forward genetics’ approach, whereby random mutagenesis of an organism’s genome was followed by metabolomics to identify specific mutant strains lacking the activity of interest. The main disadvantage of this process is the requirement to screen thousands to tens of thousands of genetic mutants in order to achieve full coverage of the test organism’s genome. Typical MS-based metabolomic strategies are not suited to high-throughput analysis (typical sample processing time >30 min 72,73). Messerli *et al* overcame this problem by initially selecting for mutants with a known phenotype that was predicted to correlate with the metabolic pathway of interest, in their case impaired starch metabolism in *Arabidopsis thaliana*. Subsequent metabolomics was then used to compare, by principal components analysis, mutants of known starch metabolizing genes with the experimentally derived mutants and subsequently to discriminate between strains with a *bona fide* deficiency in starch metabolism and those containing mutations in alternative pathways that coincidentally had pleiotropic effects on starch metabolism 74. They were thus able to identify genetic loci not previously associated with starch metabolism. Although specific metabolic activities were not linked to these genes in this case, a more detailed analysis of the metabolomics data would likely pinpoint precise metabolic deficiencies for each tested strain, as previously discussed.

**Sidebar C: In need of answers**

- How can we best prioritize large-scale metabolomic-based functional genomics efforts?
- Which organisms have a large number of orphan enzymes and are therefore good models for the discovery of new enzymatic functions and new metabolic pathways?
- Which are the most complementary methods to confirm metabolomic results?
- Which enzyme classes present the larger potential for new catalytic functions?

Defined phenotypic traits are not always associated with the presence or absence of metabolic activities of interest, nor are the global metabolic pathways to which they belong commonly known. Baran *et al* recently used a technique termed ‘metabolic footprinting’ to substantially enhance the throughput of metabolomic analysis of a library of randomly mutagenized bacterial strains of interest, for the identification of genes involved in the metabolism of test compounds 75. Transposon mutagenesis libraries of *E. coli* and *Shewanella oneidensis* (more than 8,000 mutants) were grown in media containing compounds of interest, and a shortened LC-MS protocol (2 min per sample) was then used to analyse the supernatant from spent cultures. This streamlined methodology was possible due to the substantially reduced quantity and diversity of metabolites present in the supernatant relative to cell lysates. Strains that had reduced metabolism (higher remaining levels) of the test compound of interest were analysed further to confirm the role of the target gene in the predicted metabolic pathway(s). This approach allowed the authors to assign ergothioneine histidase activity to the *S. oneidensis* gene SO3057.

## Future Direction: Imaging Mass Spectrometry

An intrinsic disadvantage of *in vitro* and *ex vivo* metabolomic experiments is the loss of information regarding the subcellular and cellular localization of the metabolites/pathways under scrutiny. For example, compartmentalized metabolite pools (such as mitochondrial versus cytoplasmic acetyl-CoA) and/or cellular heterogeneity among groups of cells in a tissue cannot be differentiated in a typical metabolomic experiment. In practical terms, the changes in abundance of putative substrates and products are diminished due to dilution of non-reacting pools of other cellular or tissue compartments. Imaging mass spectrometry (IMS) is a powerful technique that can complement traditional metabolomic experiments and provide information about spatial distribution, in addition to mass and abundance data (reviewed in 76–78). IMS techniques allow spatial mapping of metals, lipids, polar metabolites, peptides, drugs and even proteins in fixed samples, without *a priori* extraction and loss of cellular architecture. Two types of IMS methods are the most used, matrix-assisted laser desorption ionization (MALDI) imaging and secondary ion mass spectroscopy (SIMS) imaging. MALDI imaging currently has a spatial resolution of 5–50 μm, allowing analysis of single cells within tissue samples, for example. A lateral resolution under 50 nm can be achieved using SIMS, and up to five different ions can be detected simultaneously, making SIMS-based ‘ion microscopy’ a unique technique to follow the fate and organization of metabolic pathways inside and outside cells. However, with the available technology, only mono- or diatomic ions can be detected, in contrast to entire molecular ion detection obtained with MALDI imaging. Direct monitoring of new enzymatic activities with subcellular resolution is a goal that is yet to be achieved, but holds the promise to improve our understanding of cellular physiology and enzyme function at their correct compartment within cells.

## Conclusions

Enzymes control metabolism and are responsible for the formation of many of the most elaborate and interesting chemical structures in the natural world. Tools that enable rapid and unbiased functional assignment of unannotated enzymes are therefore valuable commodities in almost every field of natural science research. Metabolomics has recently emerged as a leading technology in functional genomics, where in combination with modern genetic tools and recombinant protein techniques, it has enabled a unique perspective on the identification of the mechanistic properties of test enzymes either *in vitro* or *ex vivo*. The future of metabolomics as a tool in enzyme functional assessment is inextricably linked to the future of MS as an analytical platform. As the technology improves, its reliability and accuracy as a functional genomics tool will follow suit. Similarly, methodological improvements in how metabolomics experiments are executed are constantly being sought, including increases in the breadth of metabolic diversity detected with a single analytical method 79, increases and automation in sample throughput 80, and increases in analytical sensitivity towards single-cell levels 81. Enzyme function discovery will also likely benefit from a more holistic systems approach, in which genomics, transcriptomics and proteomics will complement and validate the hypotheses and conclusions regarding protein function derived from metabolomics data sets 67. Overall, the capacity of metabolomics-based approaches to not only explore unforeseen and diverse aspects of enzyme-catalysed chemical transformations, but also associate them to global metabolic pathways and physiological functions, will likely ensure its prominence as a key technology in functional genomics strategies in the foreseeable future.

## Abbreviations

^1^^3^C, ^1^^5^N, ^1^^8^O: stable heavy isotope of specified element
ATP: adenosine triphosphate
CE: capillary electrophoresis
CoA: co-enzyme A
EC: enzyme commission
FAAH: fatty-acid amide hydrolase
FAD: flavin adenine dinucleotide
GABA: gamma-amino butyric acid
GC: gas-chromatography
HAD: haloacid dehalogenase
hP450: human cytochrome P450
IMS: imaging mass spectrometry
LC: liquid-chromatography
MALDI: matrix-assisted laser desorption ionization
MS: mass spectrometry
NAD(P)H: nicotinamide adenine dinucleotide (phosphate)
NMR: nuclear magnetic resonance
NRPS: non-ribosomal peptide synthase
PKS: polyketide synthase
SAM: S-adenosyl methionine
SIL: stable isotope labelling
SIMS: secondary ion mass spectrometry
SSA: succinic semi-aldehyde
TCA: tricarboxylic acid
TIC: total ion current
